# Secular Trends of Obesity Prevalence in Urban Chinese Children from 1985 to 2010: Gender Disparity

**DOI:** 10.1371/journal.pone.0053069

**Published:** 2013-01-08

**Authors:** Yi Song, Hai-Jun Wang, Jun Ma, Zhiqiang Wang

**Affiliations:** 1 Institute of Child and Adolescent Health, School of Public Health, Peking University, Beijing, China; 2 Centre for Chronic Disease, School of Medicine, University of Queensland, Health Sciences Building, Royal Brisbane & Women’s Hospital, Herston, Queensland, Australia; Johns Hopkins Bloomberg School of Public Health, United States of America

## Abstract

Based on the data from six Chinese National Surveys on Students Constitution and Health (CNSSCH) from 1985 to 2010, we explored the secular trend in the prevalence of obesity in urban Chinese children over a period of 25 years. The aim of this study was to examine the gender disparities in the prevalence of childhood obesity over time. The standardized prevalence of obesity in Chinese children increased rapidly during the past 25 years from 0.2% in 1985 to 8.1% in 2010. The increasing trend was significant in all age subgroups (p<0.01). Although the prevalence of obesity continuously increased in both boys and girls, the changing pace in boys was faster than that in girls. Age-specific prevalence odds ratios (PORs) of boys versus girls for obesity increased over time during the 25 year period. The prevalence of obesity in boys was significantly higher than in girls in all age-specific subgroups from 1991 and after. The gradually expanding gender disparity suggests the prevalence of obesity in boys contribute to a large and growing proportion of obese children. Therefore, it is critical for developing and implementing gender-specific preventive guidelines and public health policies in China.

## Introduction

The increasing prevalence of obesity among children and adolescents is a crisis and challenge in public health worldwide [1], [2]. The highest prevalence of childhood obesity has been observed in developed countries; however, the prevalence is increasing in developing countries as well [3]. China, with its rapid economic increase in recent three decades, has undergone epidemiological and demographic transitions affecting its population's nutritional status and created environments that contribute to increase in obesity prevalence, especially in the urban areas. The conspicuous transition included the increased availability and consumption of sugared products such as candy, soft drinks and snacks, animal protein and fat, decreased cereal intake, proliferation of fast food restaurants, and the abundance of enticements that lead to reduced physical activity, such as watching television and playing computer games [4], [5], [6]. The prevalence of childhood obesity has increased dramatically in Chinese children. Ji et al. reported that the prevalence of obesity increased from 0.13% in 1985 to 3.82% in 2005 in China [7], [8].

Although the obesity prevalence in either males or females has markedly increased in different areas of the world, there is gender difference. Generally, the prevalence of adult obesity in women is higher than that in men in most countries [9], [10], [11], [12]. However, the gender disparity in childhood obesity obviously differed from that of adult obesity in many Asia countries, such as mainland of China, India, and Turkey, where the prevalence of obesity in boys is higher than that in girls [13], [14], [15], [16], [17]. Previous studies showed the gender difference in secular trend of obesity varied in different regions, ethnicities, and populations [11], [18], [19]. However, there are no reports on how gender disparities in obesity change over time in Chinese children. Ji et al. reported there were gender differences in prevalence of childhood overweight and obesity in China, from 14.0% and 7.4% in 1985 to 34.2% and 30.3% in 2005 for males and females, respectively, but did not analyze the change of gender difference over time, i.e. whether the gender difference became larger or smaller over time [7].

The Chinese National Survey on Students' Constitution and Health (CNSSCH) has been conducted every five years since 1985, jointly launched by the Ministry of Education, the Ministry of Health, the Ministry of Science and Technology, the State of Nation Affairs, and the State Sports General Administration of People's Republic of China [13], [20], [21], [22], [23], [24]. It is, so far, the largest nationally representative sample of school-age children and adolescents in China, which provides an opportunity to study the gender disparity in secular trend of childhood obesity in China. In the present study, we used the recent 2010 CNSSCH data and previous CNSSCHs data in 1985, 1991, 1995, 2000, and 2005. The primary objective of our study was to identify whether the gender difference became larger or smaller over the past 25 years in urban Chinese school-aged children, i.e. whether the pace of increase was different for boys vs. girls. Furthermore, while Ji et al. [7] reported the changes of overweight and obesity prevalence from 1985 to 2005, having no 2010 CNSSCH data then, we used the newest 2010 CNSSCH data to show the change of obesity prevalence in recent 5 years in China.

## Subjects and Methods

This project was approved by the Medical Research Ethics Committee of the University of Queensland (#2011001199).

### Subjects

Data were obtained from the 1985, 1991, 1995, 2000, 2005 and 2010 Chinese National Surveys on Students Constitution and Health (CNSSCH) [20], [21], [22], [23], [24]. The sampling procedure, as previously described in details [13], was the same in all CNSSCH at different time points. The participants were primary and high school students aged 7∼18 years, who were selected from the same areas in each province from 1985 to 2010 with about 85% of the sampled schools remained the same in all surveys (i.e. the schools were intentionally resembled). This study only included the subjects of Han ethnicity, who accounted for 92% of the total Chinese populations, in urban areas from 26 mainland provincial capital cities and 4 municipalities, excluding Lhasa (capital city of Tibet Autonomous Region, where Han ethnicity is minority), which are all belonged to the higher socioeconomic classes of China. Although the 1991 survey was different from surveys in other years, only sampling from the higher socioeconomic classes, the data was sufficient for the analyses in the present study. All eligible participants had lived in the same area for at least one year. They had medical examination before measurement, to ensure that they had no overt physical or mental disorders. The sample sizes in CNSSCH of different years were from 4423 to 8853 in each gender- and age-specific subgroup (Table 1).

**Table 1:** Sample sizes in CNSSCH of different years.

Age group(yrs)	1985	1991	1995	2000	2005	2010
Boys						
7–9	8560	8821	4574	4799	5950	4491
10–12	8557	8853	5167	4847	6036	4497
13–15	8560	8804	5103	4834	5926	4484
16–18	8549	8838	4929	4761	5750	4450
Total	34226	35316	19773	19241	23662	17922
Girls						
7–9	8561	8835	4423	5159	5806	4496
10–12	8559	8749	4712	4996	5860	4494
13–15	8555	8774	5157	4819	6070	4489
16–18	8519	8752	5139	4725	5645	4485
Total	34194	35110	19431	19699	23381	17964

### Measures

Height (cm) and weight (kg) were measured, using the same types of instruments according to the standard procedures in all survey sites [20], [21], [22], [23], [24]. Subjects were required to wear only light clothes and stand straight, barefoot and at ease when being measured. Weight was measured to the nearest 0.1 kg with a standardized scale and height to the nearest 0.1 cm with a portable stadiometer. Both the scales and stadiometers were calibrated before use. BMI was calculated as body weight (kg) divided by height (m) squared (kg/m^2^). All measurements were conducted by a team of field professionals in each survey site. The field professionals were required to pass a training course for anthropometric measurements. Obesity was defined by using the references developed by Working Group on Obesity in China (WGOC) [25]. The children and adolescents with observed BMI≥the 95th age- and gender-specific BMI percentile value were defined as obese. For both males and females aged 18 years, those with observed BMI≥28 kg/m^2^ were considered as obese [25].

### Statistical Analyses

We estimated the prevalence of obesity (%) in different survey years according to gender and age. Standardized prevalences based on the age distribution of 1985 CNSSCH were used to compare the prevalence of obesity in different years.χ^2^ tests were used to test the prevalence differences between two adjacent years. *P*-value <0.05 was considered as statistically significant. To assess the gender and age differences at different time points, we used the logistic regression to estimate the prevalence odds ratio (POR) of gender for the prevalence of obesity in different surveys. We classified the 30 cities into 3 regions by using the same methods as the previous publication by Ji et al. [7]: (1)”North coastal region” includes 3 metropolises–Beijing, Shanghai, Tianjin, and 5 provincial capitals in north coastal region; (2)”south coastal big city” includes 7 provincial capitals in south coastal regions; and (3)”Inland big city” includes 15 inland provincial capitals. Then we performed logistic regression to estimate PORs after stratification by region. The design effect of cluster sampling by school was taken into account in the logistic regression models using Stata 12. All other analyses were conducted by using SPSS 13.0 software (SPSS, Chicago, IL).

## Results

### Trends in the Prevalence of Obesity among Urban Chinese Children

The obesity prevalence was increasing continuously over the past 25 years. Table 2 showed that the prevalence of obesity dramatically increased in both boys and girls. The standardized prevalence of obesity was 0.2%, 1.1%, 2.4%, 4.1%, 6.5% and 8.1% in 1985, 1991, 1995, 2000, 2005 and 2010 CNSSCH, respectively. In spite of the prevalence of obesity in girls was about half of that in boys in the same year, the significant increasing trend was also observed in both genders, and the significant differences were found between two adjacent years (*P*<0.01).

**Table 2:** Standardized prevalences of obesity in Chinese urban boys and girls, 1985–2010^a^.

	1985	1991	1995	2000	2005	2010
Boys	0.2	1.3**	3.0**	5.3**	8.7**	11.0**
Girls	0.1	0.9**	1.8**	3.0**	4.3**	5.2**
Total	0.2	1.1**	2.4**	4.1**	6.5**	8.1**

a Difference of prevalences between two adjacent years were examined by χ^2^ test,

** *P*<0.01.

### Trends in the Prevalence of Obesity by Age Groups

**Figure 1:**
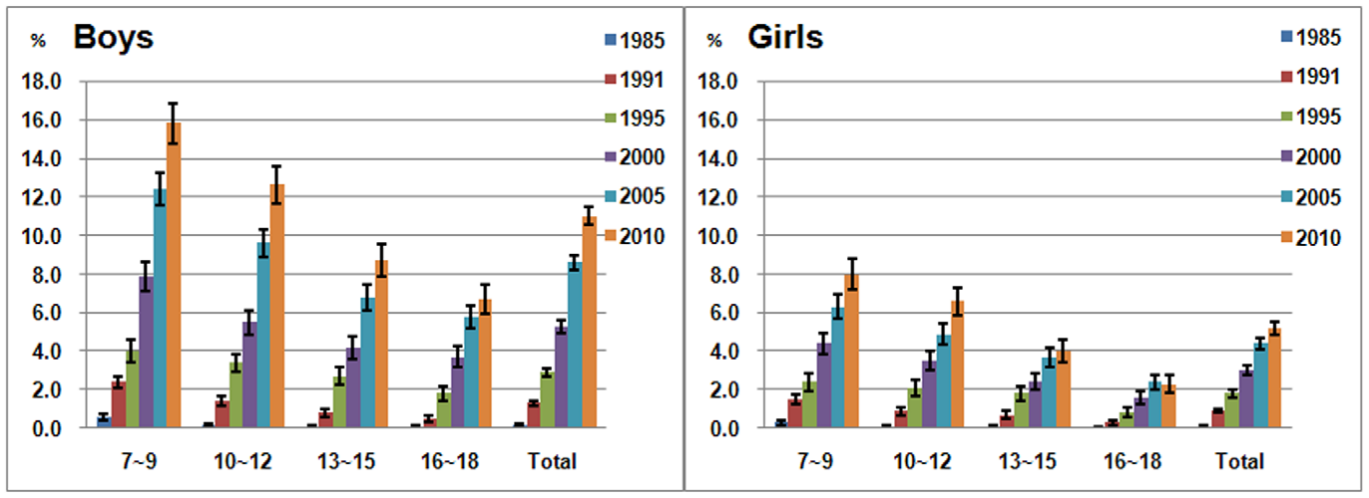
Age-specific prevalence of obesity and 95% confidence intevals (CI) in the Chinese urban boys (left figure) or girls (right figure) from 1985 to 2010. Note: In most subgroups, the increasing trends in the prevalence of obesity were statistically significant, and 95%CI of each two adjacent years were not overlapped. In recent five years, there was no significant difference in girls of 13∼15 and 16∼18 year subgroups and boys of 16∼18 year subgroup. The increasing trend was observed in both boys and girls, but the increase values in girls were less than that in boys.

As shown in Figure 1, the prevalence of obesity decreased along with age in both boys and girls, and the highest prevalence of obesity was observed in the 7 to 9 year subgroup (15.8% for boys *and* 8.0% for girls in 2010). During the past 25 years, the prevalence of obesity in all age subgroups showed an increasing trend in boys. Although an increasing trend was also observed in girls, the increase values were less than those in boys. In most gender- and age- specific subgroups, the increasing trends in the prevalence of obesity were statistically significant. However, for the most recent five years, there was no significant difference in several subgroups, including girls of 13 to 15 years and 16 to 18 years and boys of 16 to 18 years. Both the increments per year and ratio increments were higher in boys than in girls from 1991 to 2010 (See Table S1 and Table S2).

### Age-specific Prevalence Odds Ratios of Boys Versus Girls for Obesity

**Figure 2:**
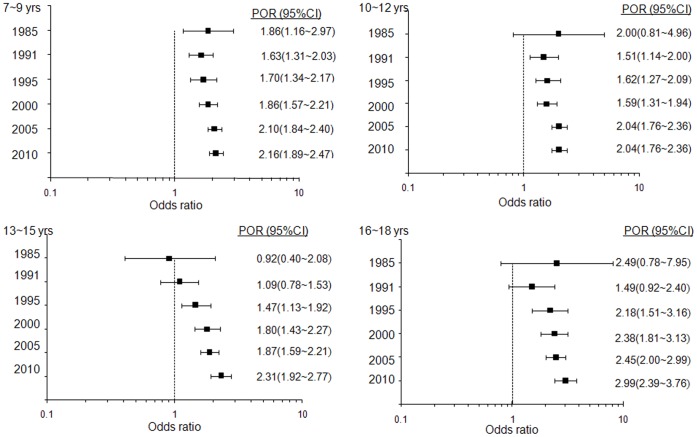
Age-specific prevalence odds ratios (POR) with 95% confidence interval (CI) for obesity of male compared with female in different years of CNSSCH. Note: In 1985, the 95% CI of POR in 10∼12, 13∼15 and 16∼18 year subgroups included 1, and in 1991, the 95% CI of POR in 13∼15 and 16∼18 year subgroups also included 1, which meant there was no significant differece in gender disparity. The PORs were significant between two genders in other subgroups, and increased over time.

As shown in Figure 1, during the past 25 years, the significantly higher increasing trend of the obesity prevalence in boys was observed in comparison with that in girls. We estimated the PORs for obesity of male compared with female in CNSSCH of different years for each age subgroups (Figure 2). In 1985 CNSSCH, there was no significant difference in the gender disparity in the 10 to 12-, 13 to 15- and 16 to 18-year subgroups, but the prevalence of obesity in boys was significantly higher than that of girls in the 7 to 9 year subgroup, OR = 1.86 (95% CI: 1.16, 2.97). In 1991, the prevalence of obesity in boys was higher than that of girls in the 7 to 9 and 10 to 12 year subgroups, with ORs of 1.63 (95% CI: 1.31, 2.03) and 1.51 (95% CI: 1.14, 2.00), respectively. The prevalence of obesity in boys was significantly higher in all age subgroups in 1995 and after. Furthermore, the PORs increased over time in all age subgroups, especially in 13 to 15- year subgroup where the POR increased from 0.92 (95% CI: 0.40, 2.08) in 1985 to 2.31 (95% CI: 1.92, 2.77) in 2010. We adjusted for correlation by school in logistic regression and found the adjusted PORs were similar to the original PORs shown in Figure 2 and Table S3. In the analyses stratified by region, the temporal trends of POR in 3 different regions were similar to the total sample (Figure S1).The results were also similar when puberty was adjusted for (data not shown).

## Discussion

We found that obesity prevalence was continuously increasing in Chinese children, and more importantly that the change of obesity in Chinese urban children was different across gender- and age-specific subgroups. To our knowledge, this is the first report in Asian children on the temporal change of gender disparity in obesity prevalence. The new contribution of our study is the finding that gender differences of obesity prevalence became larger over time (POR changes were never reported before). The 25-year period can be divided into three stages in China, each of which has special features. First, the year of 1985 can be considered as the beginning of childhood overweight and obesity epidemic in China. The prevalence of obesity was still very low at that time, and there was no gender difference in obesity prevalence in most age-specific subgroups. Second, from 1985 to 1991, there was a rapid increase in the prevalence of overweight and obesity in urban provincial capitals. The obesity prevalence in all gender- and age-specific subgroups increased 4 to 10 times. Moreover, the gender difference began to occur in 7 to 9- and 10 to 12- year subgroups. Third, after 1995, although the prevalence of obesity dramatically and continuously increased in boys of all age subgroups, the pace of obesity epidemic in girls was much slower than that in boys.

Several studies have observed that there is a gender difference in obesity prevalence, and the gender disparity in adults which have changed over time. In the United States adults, the obesity prevalence in women was continuously higher than those in men from 1960–1962 (women: 15.7%; men: 10.7%) to 1999–2000 (women: 34.0%; men: 27.7%), and the gender disparity of central obesity increased over time (1960–1962: women 19.4%, men 12.7%; 1999–2000: women 59.9%, men 38.3%) [18]. Borders et al. have determined that the gender disparity in obesity would be affected by race/ethnicity, residing types, and socioeconomic status [11]. Beydoun and his colleagues found that in the United States the mean BMI increased significantly over time and its temporal change differed across gender and ethnic groups [26]. However, little is known about gender disparity over time in Asian children.

The gender disparity in the prevalence of childhood obesity in many Western countries, i.e. the prevalence was higher in girls than boys, can be explained partly by the influence of physiological mechanism. Several studies have found that boys had significantly higher total daily energy expenditure (TDEE) and resting metabolic rate (RMR) than girls, even after adjustment for differences in size, and TDEE and RMR were significantly higher in obese children. The difference appears to be intrinsic, and contribute to the gender differences in the prevalence of obesity [27], [28].

However, in Asia countries, especially in China, where the prevalence of childhood obesity was higher in boys than girls, socio-cultural, socio-economic, behavioral, genetic factors may play some important roles in gender disparity in obesity. Chinese boys generally have different self-concept of body image compared with Western boys [29], and in accordance with the Chinese cultural value, the obesity in boys are not recognized as detrimental or unbearable. On the other hand, Chinese girls prefer a slender shape, especially during puberty, and they are more likely to control their weight compared with their male counterparts. This may explain why the prevalence of obesity in girls increased not as fast as in boys and the gender differences expanded over time, especially in the 13 to 15 and 16 to 18 year subgroups. The rapid economic development in China from a poor developing country with a GDP of only US$60 per capita in 1978 to a middle income country with a GDP of US$4700 per capita in 2010 (Exchange rate: US$1 = RMB¥6.35) in such a short time span may also have played a role in the increasing trend of obesity, as families have been able to provide sufficient foods to children [30]. The lifestyle changes may have also contributed to the gender disparity in the prevalence of obesity. There were some studies on behavioral epidemiology of the gender disparity in Chinese children. The Chinese 2005 NYRBS (National Youth Risk Behavior Surveillance) reported that nationally 4.3% of boys and 2.7% of girls had soft-drinks frequently, 23.6% of girls and 9.1% of boys tried to lose weight by restricting diet, and 29.1% of Chinese boys spent ≥2 h per day playing computer games, which were 2.0 times higher than girls [31]. The 2010 CNSSCH also investigated the obesity-related behaviors and found that unhealthy dietary habits, less physical activity and more sedentary behavior were closely related to overweight and obesity among Chinese primary and middle school students [32]. Shorter sleep time was the behavior related to gender disparity, being associated with obesity in girls not in boys [33]. In addition, gender differences in obesity prevalence may also be influenced by race and ethnicity, and the difference may be due to sex chromosome gene effects or organizational effects of gonadal hormones [34]. Wang et al. reported that gender differences existed in various obesity-related genes in Chinese children, as rs6548238 (*TMEM18*) was associated with four obesity-related indices in boys, but not in girls, in contrast rs9939609 (*FTO*) showed a strong association only in girls [35].

Our study had the following limitations. Firstly, it is not a prospective cohort study, as each cross-sectional CNSSCH was conducted on different subjects. It is possible that unintentional errors occurred when estimating the prevalence of overweight and obesity and comparing the trends. However, the CNSSCH collected nationally representative data of large sample size, and the prevalence estimated in each CNSSCH was standardized according to the age distribution of 1985 population for the purpose of comparison. It is unlikely that the observed gender differences in the prevalence of obesity could be explained by the unintentional errors. In addition, we could not adjust for some factors associated with childhood obesity, because the data on those factors were not collected in our large-scaled national surveys, including family income, parent's BMI and number of sibling. However, some publications reported that gender was an independent factor for childhood obesity when family income, parent's BMI, and the number of sibling were adjusted for [36], [37], [38]. We believe that the lacking of those factors in our surveys could not disrupt the conclusion.

Our results indicated the need of considering gender-specific approaches in the development of intervention strategies, which would improve the effectiveness of intervention and reduce obesity epidemic in China. We suggest giving special health education not only to the boys but also their parents, to transform their cultural ideas about obesity, to correct their obesity-related eating behavior, and to reduce their sedentary time. The efficacy of the gender-specific intervention awaits future studies in China. In addition, for our current understanding of the underlying complex causes of gender disparities in obesity in China is very limited, a prospective cohort study should be performed to clarify the effects of the socio-cultural, socio-economic, behavioral, genetic factors which were reported to be associated with gender disparity in obesity.

In conclusion, the prevalence of childhood obesity is still increasing in urban areas of China, and the higher prevalence in boys contributes to a large part to the increment. Our findings on gender disparity in the prevalence of obesity are critical for developing and implementing gender-specific preventive guidelines and public health policies in China and other Asian countries.

## Supporting Information

Figure S1 Prevalence odds rations (POR) with 95% confidence interval (CI) for obesity of male compared with female in different year of CNSSCH in total sample and 3 different regions.
(TIF)

Table S1 Increments per year of obesity prevalence in different stages among Chinese urban boys and girls, 1985–2010.
(DOC)

Table S2 Ratio increments of obesity prevalence between two adjacent years among Chinese urban boys and girls, 1985–2010.
(DOC)

Table S3 Age-specific prevalence odds ratio (POR) with 95% confidence interval (CI) for obesity of male compared with female in different years of CNSSCH, adjusted for correlation by school.
(DOC)

## References

[1] Lobstein T, Baur L, Uauy R (2004) Obesity in children and young people: a crisis in public health. Obes Rev 5(s1): 4–85. 15096099 10.1111/j.1467-789X.2004.00133.x

[2] Wang Y, Lobstein T (2006) World trends in childhood overweight and obesity. Int J Pediatr Obes 1: 11–25. 17902211 10.1080/17477160600586747

[3] Dehghan M, Akhtar-Danesh N, T Merchant A (2005) Childhood obesity, prevalence and prevention. Nutr J 4: 24 doi:10.1186/1475-2891-4-24. 16138930 10.1186/1475-2891-4-24 PMC1208949

[4] Cheng TO (2005) Fast food, automobiles, television and obesity epidemic in Chinese children. Int J Cardiol 98,173–174. 10.1016/j.ijcard.2004.08.019 15676189

[5] Zhai F, Wang H, Du S, He Y, Wang Z, et al.. (2009) Prospective study on nutrition transition in China. Nutr Rev 67, S56–S61. 10.1111/j.1753-4887.2009.00160.x 19453679

[6] Du S, Lu B, Zhai F Popkin BM (2002) A new stage of the nutrition transition in China. Public Health Nutr 5, 169–174. 10.1079/PHN2001290 12027281

[7] Ji C-Y, Cheng TO (2009) Epidemic increase in overweight and obesity in Chinese children from 1985 to 2005. INT J CARDIOL 132: 1–10. 18835050 10.1016/j.ijcard.2008.07.003

[8] Ma J, Cai CH, Wang HJ, Dong B, Song Y, et al.. (2012). The trend analysis of overweight and obesity in Chinese students during 1985–2010. Chin J Prev Med 46(9): 781–789 [in Chinese]. 23157879

[9] Mahfouz AA, Shatoor AS, Khan MY, Daffalla AA, Mostafa OA, et al (2011) Nutrition, Physical Activity, and Gender Risks for Adolescent Obesity in Southwestern Saudi Arabia. Saudi J Gastroenterol 17(5): 318–322. 21912058 10.4103/1319-3767.84486 PMC3178919

[10] Lovejoy JC, Sainsbury A, the Stock Conference 2008 Working Group (2009) Sex differences in obesity and the regulation of energy homeostasis. Obes Rev 10(2): 154–167. 19021872 10.1111/j.1467-789X.2008.00529.x

[11] Borders TF, Rohrer JE, Cardarelli KM (2006) Gender-specific disparities in obesity. J Community Health 31(1): 57–68. 16482766 10.1007/s10900-005-8189-8

[12] Asia Pacific Cohort Studies Collaboration (2007) The burden of overweight and obesity in the Asia–Pacific region. Obes Rev 8(3): 191–196. 17444961 10.1111/j.1467-789X.2006.00292.x

[13] Ji C-Y, Cheng TO (2008) Prevalence and geographic distribution of childhood obesity in China in 2005. INT J CARDIOL 131: 1–8. 18765165 10.1016/j.ijcard.2008.05.078

[14] Goyal RK, Shah VN, Saboo BD, Phatak SR, Shah NN, et al (2010) Prevalence of Overweight and Obesity in Indian Adolescent School Going Children: Its Relationship with Socioeconomic Status and Associated Lifestyle Factors. JAPI 58: 151–158. 20848812

[15] Unnithan AG, Syamakumari S (2008). Prevalence of Overweight, Obesity and Underweight among School Going Children in Rural and Urban areas of Thiruvananthapuram Educational District, Kerala State (India). Int J Nutr Wellness 6(2):

[16] Koçoglu G, Ozdemir L, Sümer H, Demir DA, Cetinkaya S, et al (2003) Prevalence of Obesity among 11–14 Years Old Students in Sivas-Turkey. Pakistan J Nutr 2 (5): 292–295.

[17] National Obesity Observatory (2011) International Comparisons of Obesity Prevalence. Available: http://www.noo.org.uk/NOO_about_obesity/international/Accessed 2012 May 31.

[18] Wang Y, Beydoun MA (2007) The Obesity Epidemic in the United States-Gender, Age, Socioeconomic, Racial/Ethnic, and Geographic Characteristics: A Systematic Review and Meta-Regression Analysis. Epidemiol Rev 29: 6–28. 17510091 10.1093/epirev/mxm007

[19] Gupta DK, Shah P, Misra A, Bharadwaj S, Gulati S, et al (2011) Secular Trends in Prevalence of Overweight and Obesity from 2006 to 2009 in Urban Asian Indian Adolescents Aged 14–17 Years. PLoS One 6(2): e17221. 21383840 10.1371/journal.pone.0017221 PMC3044166

[20] CNSSCH Association (1987) Report on the 1985th National Survey on Students' Constitution and Health. Beijing: People's Educational Publication [in Chinese].

[21] CNSSCH Association (1993) Report on the 1991st National Survey on Students' Constitution and Health. Beijing: Beijing Technical and Science Press [in Chinese].

[22] CNSSCH (1997) Association. Report on the 1995th National Survey on Students' Constitution and Health. Changchun: Jilin Technical and Science Publication [in Chinese].

[23] CNSSCH Association (2002) Report on the 2000th National Survey on Students' Constitution and Health. Beijing: China College & University Press [in Chinese].

[24] CNSSCH Association (2007) Report on the 2005th National Survey on Students' Constitution and Health. Beijing: China College & University Press [in Chinese].

[25] Ji CY (2005) WGOC (2005) Body mass index reference for screening overweight and obesity in Chinese school-age children. Biomed Environ Sci 18: 390–400. 16544521

[26] Beydoun MA, Wang Y (2008) Gender–ethnic Disparity in BMI and Waist Circumference Distribution Shifts in US Adults. Obesity 17: 169–176. 19107129 10.1038/oby.2008.492 PMC2610345

[27] DeLany JP, Bray GA, Harsha DW, Volaufova J (2004) Energy expenditure in African–American and white boys and girls in a 2-year follow-up of the Baton Rouge Children's Study. Am J Clin Nutr 79: 268–273. 14749233 10.1093/ajcn/79.2.268

[28] Kirkby J, Metcalf BS, Jeffery AN, O’Riordan CF, Perkins J, et al (2004) Sex differences in resting energy expenditure and their relation to insulin resistance in children (EarlyBird 13). Am J Clin Nutr 80: 430–435. 15277166 10.1093/ajcn/80.2.430

[29] Marsh HW, Hau KT, Sung RY, Yu CW (2007) Childhood obesity, gender, actual-ideal body image discrepancies, and physical self-concept in Hong Kong children: cultural differences in the value of moderation. Dev Psychol 43(3): 647–62. 17484577 10.1037/0012-1649.43.3.647

[30] National Bureau of Statistics of China (2011) China's Statistical Yearbook, 2011. Available: http://www.stats.gov.cn/tjsj/ndsj/2011/indexch.htm Accessed 2012 May 31.

[31] Ji CY, editor (2007) Report on the 2005 Chinese National Survey on Youth's Health Risk Behavior. Beijing: Peking University Medical Science Press [in Chinese].

[32] Zhang X, Song Y, Yang TB, Zhang B, Dong B, et al.. (2012). Analysis of current situation of physical activity and influencing factors in Chinese primary and middle school students in 2010. Chin J Prev Med 46(9): 781–789 [in Chinese]. 23157880

[33] Song Y, Zhang X, Ma J, Zhang B, Hu PJ, et al.. (2012). Behavioral risk factors for overweight and obesity among Chinese primary and middle school students. Chin J Prev Med 46(9): 789–796 [in Chinese]. 23157881

[34] Wisniewski AB, Chernausek SD (2009) Gender in Childhood Obesity: Family Environment, Hormones, and Genes. Gender Med 6: 76–85. 10.1016/j.genm.2008.12.001 19318220

[35] Wang J, Mei H, Chen W, Jiang Y, Sun W, et al (2012) Study of eight GWAS-identified common variants for association with obesity-related indices in Chinese children at puberty. Int J Obes 36(4): 542–547. 10.1038/ijo.2011.218 22083549

[36] Martin KS, Ferris AM (2007) Food insecurity and gender are risk factors for obesity. J Nutr Educ Behav 39(1): 31–6. 17276325 10.1016/j.jneb.2006.08.021

[37] Babey SH, Hastert TA, Wolstein J, Diamant AL (2010) Income disparities in obesity trends among California adolescents. Am J Public Health 100(11): 2149–55. 20864702 10.2105/AJPH.2010.192641 PMC2951974

[38] Keane E, Layte R, Harrington J, Kearney PM, Perry IJ (2007) Measured Parental Weight Status and Familial Socio-Economic Status Correlates with Childhood Overweight and Obesity at Age 9. 7(8): e43503. 10.1371/journal.pone.0043503 PMC3422292 22912886

