# Supplementary figures and images for: Secular Trends of Obesity Prevalence in Urban Chinese Children from 1985 to 2010: Gender Disparity
Source: PLoS One. 2013 Jan 8;8(1):e53069. doi: 10.1371/journal.pone.0053069 (PMC3540080; doi:10.1371/journal.pone.0053069)

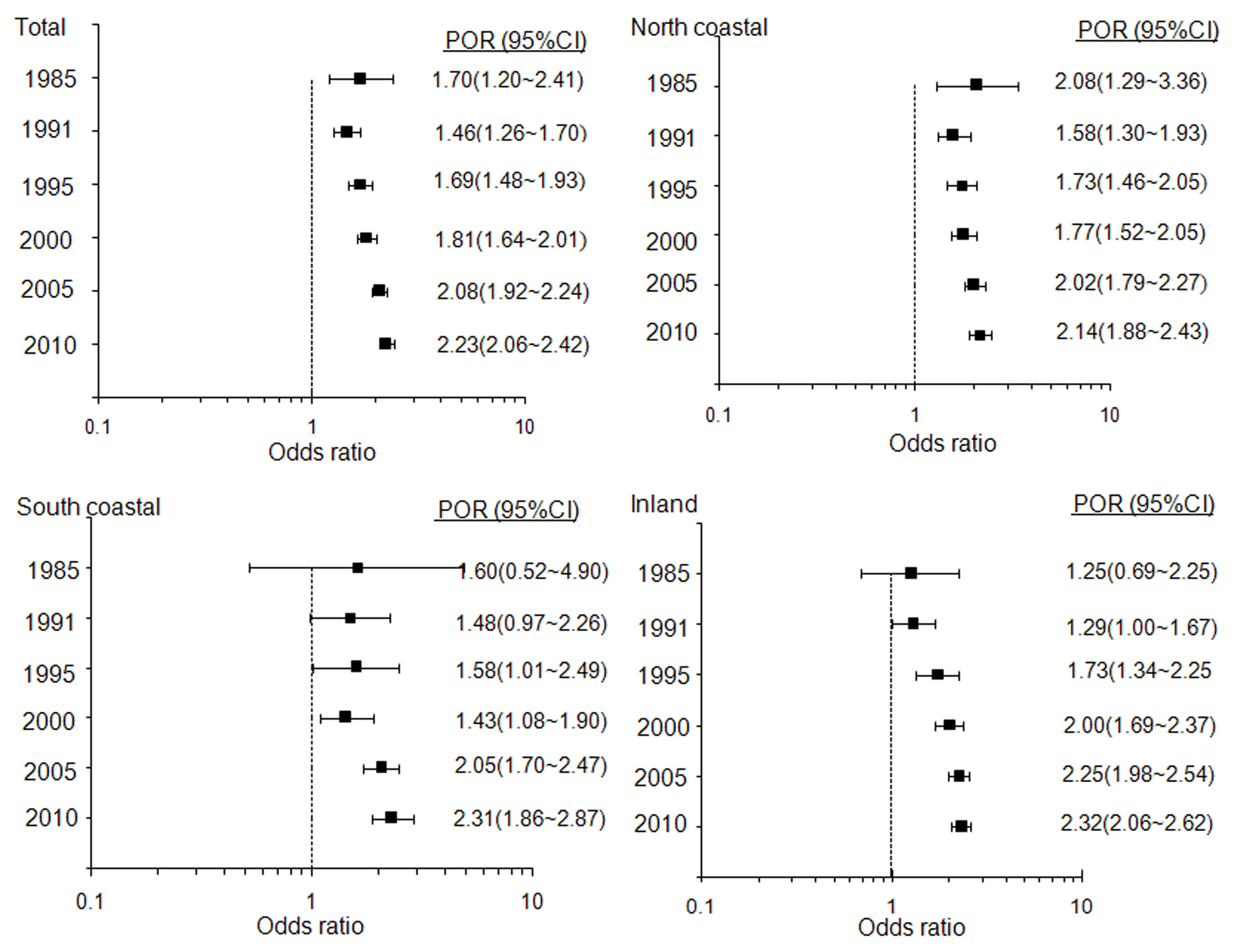

Supplement: Figure S1 — Prevalence odds rations (POR) with 95% confidence interval (CI) for obesity of male compared with female in different year of CNSSCH in total sample and 3 different regions. (TIF) [file pone.0053069.s001.tif]
