# Supplementary material for: Secular Trends of Obesity Prevalence in Urban Chinese Children from 1985 to 2010: Gender Disparity
Source: PLoS One. 2013 Jan 8;8(1):e53069. doi: 10.1371/journal.pone.0053069 (PMC3540080; doi:10.1371/journal.pone.0053069)
Supplement: Table S1 — Increments per year of obesity prevalence in different stages among Chinese urban boys and girls, 1985–2010. (DOC) [file pone.0053069.s002.doc]

Table S1 Increments per year of obesity prevalence in different stages among Chinese urban boys and girls, 1985-2010

|  | Increment/year（1985~1991） | Increment/year（1991~1995） | Increment/year（1995~2000） | Increment/year（2000~2005） | Increment/year（2005~2010） |
| --- | --- | --- | --- | --- | --- |
| Boys | 0.2 | 0.4 | 0.5 | 0.7 | 0.5 |
| Girls | 0.1 | 0.2 | 0.2 | 0.3 | 0.2 |
| Total | 0.2 | 0.3 | 0.3 | 0.5 | 0.3 |
