# Supplementary material for: Secular Trends of Obesity Prevalence in Urban Chinese Children from 1985 to 2010: Gender Disparity
Source: PLoS One. 2013 Jan 8;8(1):e53069. doi: 10.1371/journal.pone.0053069 (PMC3540080; doi:10.1371/journal.pone.0053069)
Supplement: Table S2 — Ratio increments of obesity prevalence between two adjacent years among Chinese urban boys and girls, 1985–2010. (DOC) [file pone.0053069.s003.doc]

Table S2 Ratio increments of obesity prevalence between two adjacent years among Chinese urban boys and girls, 1985-2010

|  | 1991/1985 | 1995/1991 | 2000/1995 | 2005/2000 | 2010/2005 |
| --- | --- | --- | --- | --- | --- |
| Boys | 6.5 | 2.3 | 1.8 | 2.9 | 1.3 |
| Girls | 9.0 | 2.0 | 1.7 | 2.4 | 1.2 |
| Total | 5.5 | 2.2 | 1.7 | 2.7 | 1.2 |
