# Supplementary material for: Secular Trends of Obesity Prevalence in Urban Chinese Children from 1985 to 2010: Gender Disparity
Source: PLoS One. 2013 Jan 8;8(1):e53069. doi: 10.1371/journal.pone.0053069 (PMC3540080; doi:10.1371/journal.pone.0053069)
Supplement: Table S3 — Age-specific prevalence odds ratio (POR) with 95% confidence interval (CI) for obesity of male compared with female in different years of CNSSCH, adjusted for correlation by school. (DOC) [file pone.0053069.s004.doc]

Table S3 Age-specific prevalence odds ratio (POR) with 95% confidence interval (CI) for obesity of male compared with female in different years of CNSSCH, adjusted for correlation by school

| Age group（yrs） | Obesity | |  | Obesity adjusted for correlation by school | |
| --- | --- | --- | --- | --- | --- |
|  | OR | 95%CI |  | OR | 95%CI |
| 7~9 |  |  |  |  |  |
| 1985 | 1.86 | 1.16~2.97 |  | 1.86 | 1.16~2.99 |
| 1991* | 1.63 | 1.31~2.03 |  | - | - |
| 1995 | 1.70 | 1.34~2.17 |  | 1.70 | 1.29~2.24 |
| 2000 | 1.86 | 1.57~2.21 |  | 1.91 | 1.47~2.47 |
| 2005 | 2.10 | 1.84~2.40 |  | 2.10 | 1.85~2.38 |
| 2010 | 2.16 | 1.89~2.47 |  | 2.16 | 1.87~2.50 |
| 10~12 |  |  |  |  |  |
| 1985 | 2.00 | 0.81~4.96 |  | 2.00 | 0.83~4.86 |
| 1991* | 1.51 | 1.14~2.00 |  | - | - |
| 1995 | 1.62 | 1.27~2.09 |  | 1.62 | 1.29~2.05 |
| 2000 | 1.59 | 1.31~1.94 |  | 1.50 | 1.17~1.94 |
| 2005 | 2.04 | 1.76~2.36 |  | 2.04 | 1.67~2.48 |
| 2010 | 2.04 | 1.76~2.36 |  | 2.03 | 1.68~2.48 |
| 13~15 |  |  |  |  |  |
| 1985 | 0.92 | 0.40~2.08 |  | 0.92 | 0.40~2.07 |
| 1991* | 1.09 | 0.78~1.53 |  | - | - |
| 1995 | 1.47 | 1.13~1.92 |  | 1.47 | 1.07~2.03 |
| 2000 | 1.80 | 1.43~2.27 |  | 1.86 | 1.39~2.48 |
| 2005 | 1.87 | 1.59~2.21 |  | 1.87 | 1.59~2.21 |
| 2010 | 2.31 | 1.92~2.77 |  | 2.31 | 1.97~2.71 |
| 16~18 |  |  |  |  |  |
| 1985 | 2.49 | 0.78~7.95 |  | 2.49 | 0.75~8.23 |
| 1991* | 1.49 | 0.92~2.40 |  | - | - |
| 1995 | 2.18 | 1.51~3.16 |  | 2.18 | 1.47~3.22 |
| 2000 | 2.38 | 1.81~3.13 |  | 2.43 | 1.62~3.64 |
| 2005 | 2.45 | 2.00~2.99 |  | 2.45 | 1.98~3.01 |
| 2010 | 2.99 | 2.39~3.76 |  | 2.99 | 2.48~3.61 |

* The school ID were not available in 1991 year survey, so that we could not adjust for this variable in 1991.
